# [Retracted] Overexpression of SHP2 tyrosine phosphatase promotes the tumorigenesis of breast carcinoma

**DOI:** 10.3892/or.2026.9081

**Published:** 2026-02-24

**Authors:** Zhongqian Hu, Haoshu Fang, Xinyi Wang, Danlei Chen, Zhuo Chen, Siying Wang

Oncol Rep 32: 205–212, 2014; DOI: 10.3892/or.2014.3201

Following the publication of the above article and a corrigendum (doi: 10.3892/or.2017.5455) that was issued in 2017 to address issues of incorrect data assembly in Figs. 3 and 7, an interested reader drew to our attention that data also appeared to be duplicated in Fig. 4C, and data featured in Figs. 2 and 5 subsequently appeared in an article published by the same research group in the journal *Oncotarget*. Additionally, following an independent re-evaluation of the data in this paper made by the Editorial Office, duplicated data were also noted in Fig. 5A, and some of the data in question subsequently appeared in an article in *Journal of Enzyme Inhibition and Medicinal Chemistry* in 2022 written by different authors at different institutes, which has since been retracted.

In view of these additional findings, even though the possibility of a further corrigendum was originally considered, the Editor of *Oncology Reports* has decided that the paper should be retracted on account of a lack of confidence in the presented data. The authors were asked for an explanation to account for these concerns, but the Editorial Office did not receive a satisfactory reply. The Editor apologizes to the readrship for any inconvenience caused.

